# High frequency GPS bursts and path-level analysis reveal linear feature tracking by red foxes

**DOI:** 10.1038/s41598-019-45150-x

**Published:** 2019-06-20

**Authors:** Richard Bischof, Jon Glenn Omholt Gjevestad, Andrés Ordiz, Katrine Eldegard, Cyril Milleret

**Affiliations:** 1 Faculty of Environmental Sciences and Natural Resource Management, Norwegian University of Life Sciences, Høgskoleveien 12, 1432 Ås, Norway; 2 Faculty of Science and Technology, Norwegian University of Life Sciences, PO Box 5003, NO-1432 Ås, Norway

**Keywords:** Ecology, Behavioural ecology

## Abstract

There is a need to quantify and better understand how wildlife interact with linear features, as these are integral elements of most landscapes. One potentially important aspect is linear feature tracking (LFT), yet studies rarely succeed in directly revealing or quantifying this behavior. In a proof-of-concept study, we employed short-term intensive GPS monitoring of red foxes (*Vulpes vulpe*s) in a multiple-use landscape in southern Norway. Using periodic bursts of high frequency GPS position fixes, we performed modified path selection analyses to estimate the propensity of foxes to track natural and man-made linear features (roads, forest edges, and streams) once they are encountered. Foxes in our study tracked primarily forest edges and roads. Forty-three percent of bursts that encountered any linear feature resulted in LFT. LFT, although prominent, was manifested as a short-lived behavior, with overall median times to linear feature abandonment around two minutes. Movement speeds were highest along roads, perhaps due to greater ease of travel or higher perceived risk. In the highly heterogeneous habitats that characterize human-dominated landscapes, LFT may be manifested at such a fine spatio-temporal scale that it would remain hidden during telemetry studies employing conventional position fix frequencies. The approach described here may aid others studying spatial behaviors that are manifested over very short durations, yet are biologically significant.

## Introduction

Linear features of both man-made and natural origin crisscross most of Earth’s terrestrial surfaces. Habitat edges, rivers, and transportation networks are conspicuous attributes of most landscapes^1,2^. In human dominated landscapes, they affect wildlife habitat use^3^, movements and dispersal^4,5^, daily activity patterns^6^, gene flow^7,8^, foraging^9,10^, and survival^11,12^. These effects scale up to impacts on the dynamics, distributions, and genetic makeup of populations, ultimately influencing trophic interactions^13–15^.

Due to potentially profound consequences for management and conservation^16,17^ and in the face of continued human development, there is a need to better understand how wild animals interact with linear features in their environment^18,19^. One interesting, yet underexplored, aspect is travel along linear features, i.e., when animal movement paths coincide with distinctive linear ground features. Linear feature tracking (hereafter, LFT) may for instance be motivated by increased movement efficiency^10^, signaling/scent marking^20^, and foraging opportunities^21^. Linear features can serve as corridors during the dispersal process of both plants^22^ and animals^23,24^. Nevertheless, LFT can have inadvertent negative effects on survival^11,12^, which may eventually lead to ecological traps^25^. LFT may also make habitat use more predictable, which can be exploited during ecological studies and wildlife monitoring, for example using camera trapping along trails^26^. Indeed, use of linear features has repeatedly been reported, or at least inferred, for medium and large carnivores^10,27–30^. These highly mobile species may follow linear features such as roads or habitat edges to efficiently travel through the landscape^10^ and for the foraging opportunities they offer^28^.

Most studies explore selection for sites on or in proximity to linear structures; few have tested for or quantified actual movement along them^30^. There are alternative approaches to quantifying use/selection of linear features (camera trapping, noninvasive genetic sampling, signs surveys/snow tracking^20,26^), but most published studies rely on GPS telemetry data to make inferences^10^. Studying LFT through telemetry applications requires 1) position data of sufficient spatio-temporal resolution given the study species’ movement characteristics and the degree of landscape heterogeneity, 2) correspondingly detailed spatial data on landscape features potentially involved in LFT, and 3) analytical approaches that allow investigators to test for and quantify the propensity of individuals or populations to perform LFT. Our intention with the present study is to demonstrate an approach collecting and analyzing telemetry data when investigating LFT and other behaviors manifested at fine spatio-temporal scales.

Position error aside, the spatio-temporal grain of GPS telemetry is determined by the frequency of position fixes. Habitat selection is a hierarchical process occurring at multiple scales^31^. Depending on the scale at which linear feature tracking is manifested, typical GPS position fix intervals in the order of hours may yield position data of sufficient resolution to quantify apparent associations with linear features, but would not allow identifying LFT as such. For example, a schedule of one GPS position fix every five minutes may suffice to pick up the tracking of low-tortuosity seismic lines in Canada by wolves (*Canis lupus*^27^), a species known for routine long-distance movement. Fixes every half an hour or every hour served to find resting sites of brown bears (*Ursus arctos*^32^). However, even a 5-min inter-fix interval may be too long to detect and describe LFT behavior of species inhabiting fragmented rural or urban landscapes with an abundance of tortuous and intersecting linear features of multiple types.

Most telemetry studies face the challenge of managing the tradeoff between frequency (grain) and longevity (extent) of position data^33^. One way to achieve finer temporal grain is to reduce the inter-fix interval. In domestic animals such as pets and livestock and in humans, the utility of GPS tracking systems is largely dependent on the precision and temporal detail of localization, the latter being influenced by the sampling rate^34,35^. Unlike these cases, in which batteries can easily be replaced or recharged at the users’ convenience, reducing inter-fix interval in wildlife studies comes at the cost of reduced collar operating life due to the accelerated power consumption. Assuming a linear relationship between the total number of position fixes and cumulative power consumption (which is a generous assumption), in the case of a GPS collar with a 1-year operating life at an inter-fix interval of 1 hour, a reduction of the inter-fix interval to 1 minute would cut the collar’s operating life to 6 days. While this may be acceptable if the focus of the entire study is on detailed movements during a short time period, such a strategy may not be justifiable in the case of studies that want to draw inferences at multiple temporal scales and when capture and handling are logistically challenging and costly^36^.

Here, we use periodic bursts of high frequency GPS position fixes^34,37,38^ to balance the need for scale-transcendent information and battery life. Inter-burst intervals can be chosen to yield information about behavior occurring at larger spatial and temporal scales, e.g., within the home range, while a series of rapid consecutive GPS position fixes within each burst allows inference about behavior at finer scales, e.g., within specific habitat patches. The utility of GPS position information with a high temporal resolution is boosted by the increasing availability of high-definition spatial data, for example from remote sensing applications^39^.

**Figure 1:**
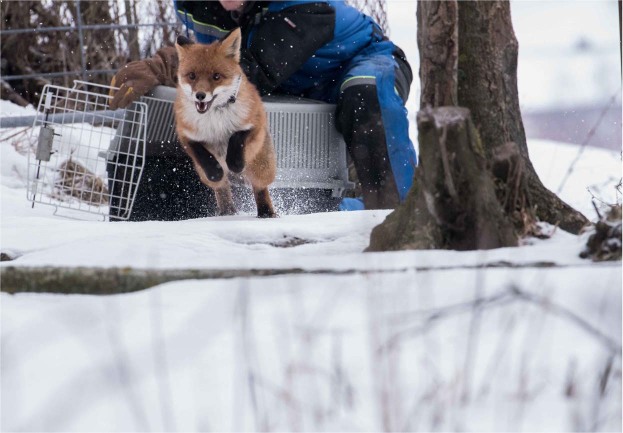
Release of a red fox (Vv2) following capture and GPS-collaring. Photo: C. Milleret.

**Figure 2:**
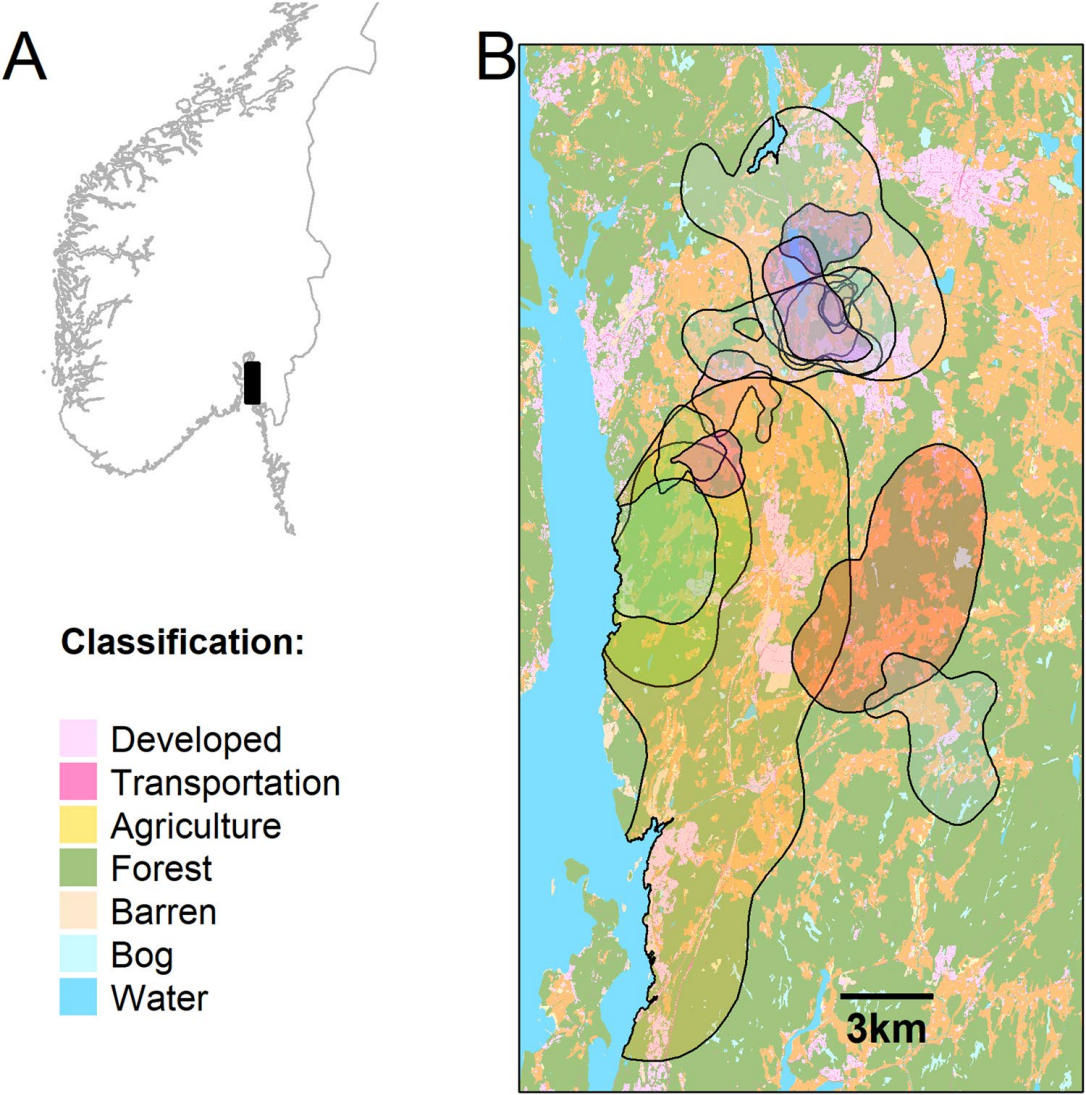
Maps of the study area in southern Norway (**A**), with polygons indicating 90% kernel vertices based on multi-day positions of 17 foxes (different shades for different individuals; (**B**). One additional female (Vv5; not shown here) dispersed south of the study area immediately after tagging. Maps were created in R version 3.5.1^44^ (www.R-project.org).

As a proof-of-concept study, we conducted short-term, high-intensity GPS monitoring of 18 red foxes (*Vulpes vulpe*s; 7 females, 11 males, Figs 1 and 2) in a mosaic landscape in southern Norway. Globally, the red fox is the most widely distributed and, arguably, most adaptable carnivore species, often inhabiting and thriving in human-dominated landscapes^40,41^. Using periodic (every 10–20 minutes) bursts of GPS positions with approximately 15 second intervals between fixes, we collected position data of high spatio-temporal resolution. We performed a series of analyses to quantify LFT associated with three linear features: roads, streams, and open/closed habitat boundaries (forest edges). We asked the following questions:

1. Linear feature tracking (LFT): If a linear structure is encountered, do foxes track it or stay near it? Prediction: Conditional on encountering linear features, foxes may track these features. The propensity to perform LFT will depend on linear feature type and vary between individuals.
2. Duration of LFT (${t}_{{LFT}}$): If LFT occurs, how long does it last? Prediction: LFT is a short-lived phenomenon (in the order of minutes) when performed by foxes in a heterogeneous, fragmented environment.
3. Movement speed during LFT ($${v}_{{LFT}}){:}$$ Does linear feature type influence the speed of movement during LFT? Prediction: Movement speeds will be higher along roads than other linear features due to the greater ease of travel, but also greater risk of, and exposure to, human disturbance.

The high spatio-temporal resolution yielded by the telemetry bursts, in combination with path-level analysis, allowed us to detect LFT and individual heterogeneity therein, which would have remained obscured or entirely hidden from investigations using conventional position intervals.

## Methods

### Study system

The study was carried out in southern Norway, in the municipalities Vestby (59°34.0′–59°38.3′N, 10°38.8′–10°43.5′E) and Ås (59°39.6′–59°42.7′N, 10°43.8′–10°47.7′E), at elevations 0–152 m.a.s.l (Norwegian Mapping Authority 2018; https://norgeskart.no/). In addition, one of the foxes moved south of this core study area, reaching Fredrikstad (59°13′N–10°56′E). About 50% of the study area is covered by forest managed for timber or firewood production (Statistics Norway 2017; https://www.ssb.no/), mainly mixed conifer-deciduous boreal forests. The dominating tree species are Norway spruce *Picea abies* and Scots pine *Pinus sylvestris*, whereas birch *Betula* spp. are the most common deciduous trees, followed by rowan *Sorbus aucuparia*, *Salix* spp. and European aspen *Populus tremula*. The entire landscape is influenced by human activities and land use (Fig. 2), with 31% cultivated land, 18% of other open areas and a high road density. Human population density is 159 residents/km^2^, mainly concentrated in small town centres or residential areas, but also on farms and single houses scattered throughout the so-called “cultural landscape”. The result is a fragmented landscape with forest patches of varying sizes interspersed with crop fields, pastures, and human settlements. In addition to linear landscape features like forest edges and streams, purely anthropogenic linear elements like roads are common. Fox hunting is legal in the study area throughout the year except during the season when females are with dependent young (April 15 – July 15). On average, 0.5 foxes/km^2^/year were killed in 2013–2017 in the study area (Statistics Norway 2017; https://www.ssb.no/). The climate is Temperate-Continental with an average temperature in the coldest month (February) of −1.9 °C/−4.7 °C in 2017/2018 (Norwegian Metrological Institute 2018; https://www.met.no/).

### GPS collars

We developed and built custom GPS devices, specifically designed using off-the-shelf components together with a customized printed circuit board and a standard micro controller. The backbone of the electronics is a SIM808 GSM/GPRS module from SimCom™ featuring built in GPS, General Packet Radio Service (GPRS) and Bluetooth functionality. This module is controlled by an 8 bit ATmega 328p micro controller running our own software, optimized for the application with main focus on robustness and reliability in harsh environments. Once active, the collar can be fully controlled by a predefined subset of commands through standard SMS messages, thus allowing any type of basic cellphone to be used as a communication device. The commands range from requesting current battery level and signal strength to changing the sampling/burst rates and the default behavior when GSM and/or GPS signals are lost.

Raw data were sent immediately over GPRS to a dedicated server and stored. GPS units and batteries (lithium polymer; 3000mAh) were housed in 3D-printed plastic cases (7 cm × 4 cm × 4 cm), which were attached to a collar made of a 2 cm wide and 1 mm thick plastic coated strap (BioThane®). A short cotton string was incorporated into each collar as a wear-and-tear-type drop off mechanism. Assembled GPS collars weighed on average 123 g, which was less than 2.3% of the average fox body weight (5.3 kg) in our study.

### GPS position error determination

In order to quantify the true position error of our custom-built GPS devices during movement, we conducted empirical tests using a high-end APX-15 GNSS/IMU unit. The reference unit was mounted on the roof of a car together with two different GPS collars, and a test route was driven on narrow forest roads with varying canopies and at slow speed (1–5 m/s). Due to the inertial measurement unit (IMU) the reference unit is able to maintain its high accuracy at the level of a few centimeters even when the main GNSS antenna is temporarily obstructed. The raw data collected from the reference unit were stored and post processed for the whole trajectory yielding an overall precision for the reference trajectory of about 10 cm. The median true error of our GPS collars was 2.4 m (95% confidence interval, hereafter CI: 0.6 m–10.1 m), based on a sample of 300 positions.

### Fox capture and handling

Foxes were captured in large wooden box traps (approximately 200 cm × 80 cm × 80 cm) with two trapdoors, which are legal and standard traps in Norway for live capture of small and medium-sized carnivores. Traps were baited with meat (primarily dead chickens *Gallus gallus domesticus*) and monitored daily. Captured foxes were restrained with the help of gloves, a catch pole, and/or neck tongues. In addition to GPS collar attachment, we recorded the sex and weight of each fox, and collected hair samples as voucher specimen and for future DNA analysis. Handling, between removal from the trap and release (Fig. 1) lasted between 10–20 min. The animals’ eyes were covered with a dark cloth during handling to reduce stress, as handling was performed without anesthesia. All capture and handling conformed to the current laws and regulations in Norway and the study was approved (case IDs 2016/4769 and 18/211316) by the Norwegian Animal Research Authority (FOTS) under the auspices of the Norwegian Food Safety Authority.

### GPS tracking and data preparation

GPS collars were programmed to take position fixes in bursts of 20 (15 second inter-fix interval), with an inter-burst interval of 10–20 minutes. The inter-burst interval increased to 60 minutes once the battery had lost approximately 60% of its original charge. Positions were transmitted immediately after collection via GSM network to an internet server, from which they were downloaded for processing and analysis. To minimize the potential initial effects of capture and handing on movement and behavior, we excluded positions collected during the first day after each fox was released. Furthermore, since we were interested in position data collected during movement/travel, we removed those portions from the data that indicated stationary or localized behavior, e.g., time in dens and day beds or time spent investigating focal points. For each burst, we calculated sinuosity^42,43^ and average movement speed between consecutive positions. We then excluded all bursts with sinuosity >0.5 and average movement speed <0.1 m/s. Data preparation and subsequent analyses were conducted using R version 3.5.1^44^.

### Linear features

**Figure 3:**
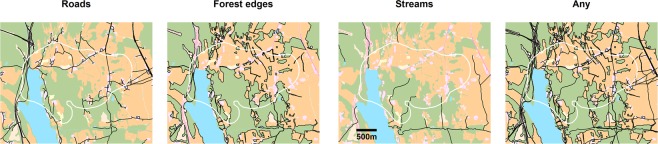
Example of the spatial configuration of the three linear feature types (roads, forests and streams) within the area used by one fox (Vv4; 90% kernel; white dashed outline). The panel to the right shows all three linear feature types combined. Background colors represents paler versions of the land cover classification described in Fig. 2. Maps were created in R version 3.5.1^44^ (www.R-project.org).

Forest edges, roads, and streams are the most prominent linear features in our study area (in that order of prevalence) and were therefore selected in our test for LFT. We utilized contemporary (2016) high-resolution (5 m) land cover data (AR5; https://www.nibio.no/tema/jord/arealressurser/arealressurskart-ar5) to identify edges between forest and other (primarily open) land cover types (Fig. 3). We obtained GIS data of streams and roads through the Norwegian Mapping Authority’s geographic data portal (kartkatalog.geonorge.no).

### Linear feature tracking (LFT)

**Figure 4:**
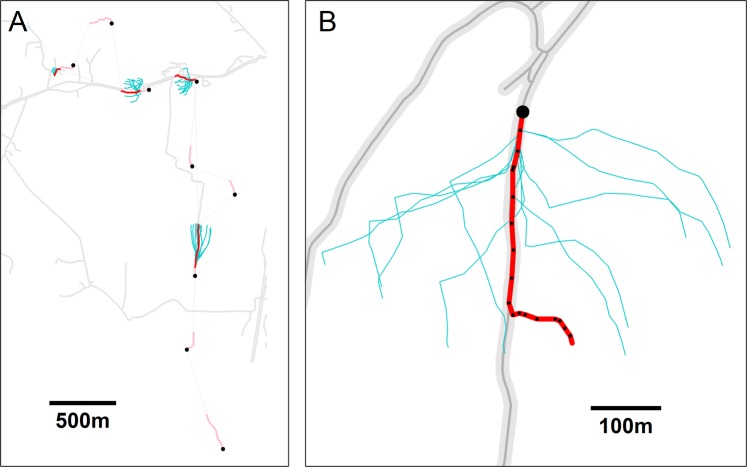
Examples illustrating the null model approach for the analysis of linear feature tracking (LFT) by red foxes. (**A**) Random trajectories (turquoise lines; based on empirical distributions of step lengths and bearings) for bursts following encounters with a linear feature (roads; grey lines). Black dots denote the start of the trajectory. Pink and red lines indicate burst trajectories before and after the initial encounter with the linear feature, respectively. (**B**) Close-up of a burst with apparent LFT and associated random trajectories (turquoise lines) as in A). Black dots are GPS positions, the largest one indicating the start of the burst.

We used a modified burst-level path selection analysis to quantify the propensity of individuals to stay in close proximity to linear features once these had been encountered. We first selected bursts with at least one position within 10 m of a linear feature (i.e., road, forest edge, or stream). This proximity buffer width was chosen in order to balance the need to capture truly close association with the linear feature, i.e., the animal was either walking directly on the feature or right next to it, with the GPS position error (upper 95%CI limit of 10.1 m). We then considered all positions in a burst following and including the first position within the proximity buffer as an observed (“case”) trajectory. For each case trajectory, we simulated 10 random trajectories (“control”) with step lengths and relative turning angles sampled from the empirical distributions of these parameters from the respective case trajectory (Fig. 4). This represents a null model based on a correlated random walk^45^. We then fit conditional logistic regression models (R package “survival”^44,46^) to the resulting data with observation type (case vs. control) as the response and whether a position was located within 10 m of a linear feature or not as the predictor. This approach tests whether the true trajectory taken by the fox is more or less likely to exhibit LFT than alternative realizations of the true trajectory. We repeated the analysis with buffer widths of 5 m and 15 m, without qualitative changes to the main results. All analyses were performed in R version 3.5.1^44^.

Because linear features may co-occur (e.g., roads through forests also represent forest edges), all linear features were included as dummy covariates in the same model (coded 0/1 for inside and outside the distance threshold, respectively). As a result, any observation (case and control) could be placed in various combinations of overlapping linear feature buffers or outside all buffer. For simplicity, we chose to use only additive effects, but this approach could also be used to accommodate interactions. Burst ID (i.e., case trajectory ID) was included as a stratum^47^ and the sequence ID of a position as a clustering variable. Separate models were fitted for each individual fox as we were interested in individual differences in the propensity of performing LFT and because models differed between individuals (not all individuals encountered all linear feature types), thus precluding the commonly used two-step approach^48^. In each individual model, we included only those linear features types that were encountered during at least 5 different bursts. Animals may perform continuous LFT by stitching together movement along multiple feature types. Therefore, in addition to the models that differentiated between linear feature types, we also ran models with roads, forest edges, and streams combined into one linear feature (“any”, Fig. 3).

Our approach to path-level selection analysis differs from most applications of path analysis, which generally employ random rotation and shifting of empirical paths for null model generation^49–53^. We chose the correlated random walk, based on empirical distributions of turning angles and distances, to generate our null model (Fig. 4). In our opinion, this more closely reflects the process leading to realized path topology as a sequence of small-scale movement choices, rather than a predetermined fixed path shape. Furthermore, GPS positions were obtained every 15 seconds during bursts. At this scale, spatial autocorrelation is very high and pure step selection is not a meaningful test of LFT. Most individual steps are short and, in the case of trajectories closely tracking the line, most or all simulated control steps may fail to leave the LFT buffer around the linear feature.

Association with linear features indicates possible LFT, but, even at the fine temporal scale of our data, it is not direct proof of it. Individuals may stay close to linear features by moving along them, but could also perform other behaviors near a linear feature without necessarily tracking it. In order to quantify the incidence of LFT from the GPS data and describe attributes of LFT, we defined an LFT event as a contiguous sequence of positions that a) all fell within the 10 m proximity buffer, b) consisted of at least 3 positions, c) represented a total movement distance of at least 35 m, and d) exhibited displacement-to-distance ratio of >0.5. The displacement-to-distance ratio was calculated as the average displacement of all subsequent points from the first position in a sequence divided by the average distance moved between points. Setting a threshold on this measure removed position sequences within the buffer that represented movement without apparent direction rather than actual travel.

### Duration of LFT (${{\boldsymbol{t}}}_{{\boldsymbol{LFT}}}$)

We used time-to-event analysis^54^ to estimate the duration of LFT events on a given linear feature type. Although distance to event analysis^55^ is a feasible alternative for quantifying the endurance of LFT, we were interested in relating the results to the temporal component (schedule) of the GPS position fix strategy. Only position sequences identified as LFT (see above) were included in this analysis. An event (departure from LFT) was defined as the instance when the LFT sequence was followed by a position within the same burst that exceeded the 10 m distance threshold (event = 1). LFT sequences that were not abandoned before the end of the burst were right censored, i.e., these LFT sequences were considered available for LFT abandonment during analysis, but the event was considered to have occurred after the observation period. The time variable was defined as the number of seconds from the first position of a sequence until either censoring or departure occurred. In order to further minimize bias in time-to-event estimates, we excluded LFT events for which the starting point was unknown, i.e., when the first point in the burst was already within the buffer. We then constructed Kaplan-Meier time-to-event curves for each linear feature type and estimated median time-to-event, i.e., the time by which 50% of LFT events had not yet ended, using the survfit function in the R package “survival”^46^. We used bootstrapping to derive 95% CI around median times-to-event, sampling at both the individual and burst level to account for non-independence. Although median time to event is directly linked with the proximity threshold, patterns in relative hazard (differences in the instantaneous potential of abandoning/ending an LFT event) with respect to feature type are unlikely to change drastically as long as the distances picked represent a reasonably close proximity to the linear feature. We used Cox proportional hazards models using the coxph function in the R package “survival”^46^ to test for differences in the time-to-event associated with different feature types, with feature type as the predictor variable, individual ID as strata variables, and burst ID as cluster variables.

### Movement speed during LFT (${{\boldsymbol{v}}}_{{\boldsymbol{LFT}}})$

We calculated average feature-dependent travel speeds for each fox based on all LFT events identified on or along a given linear feature type. Ninety-five percent confidence limits were derived by bootstrapping. We used linear mixed regression with R package lme4^56^ to test for and quantify differences in speeds along different linear feature types, with the log-transformed average speed during an LFT event as the response, feature type (roads, forest edges, and streams) as fixed effect, and burst ID nested within individual ID as random effect.

## Results

### GPS tracking

Eighteen foxes (7 females; 11 males) were GPS tagged during this study, including six that could be classified as juveniles based on size and appearance (Supplementary Table S1). All foxes were GPS tracked for a short duration (5–28 days), but with high intensity (1123–4617 positions per fox). Depending on the individual, between 35% and 94% of positions (after cleaning and removal of the first 24hrs after marking) were categorized as “traveling” based on sinuosity and speed and thus included in the analysis (Supplementary Table S1).

### Linear feature tracking (LFT)

**Figure 5:**
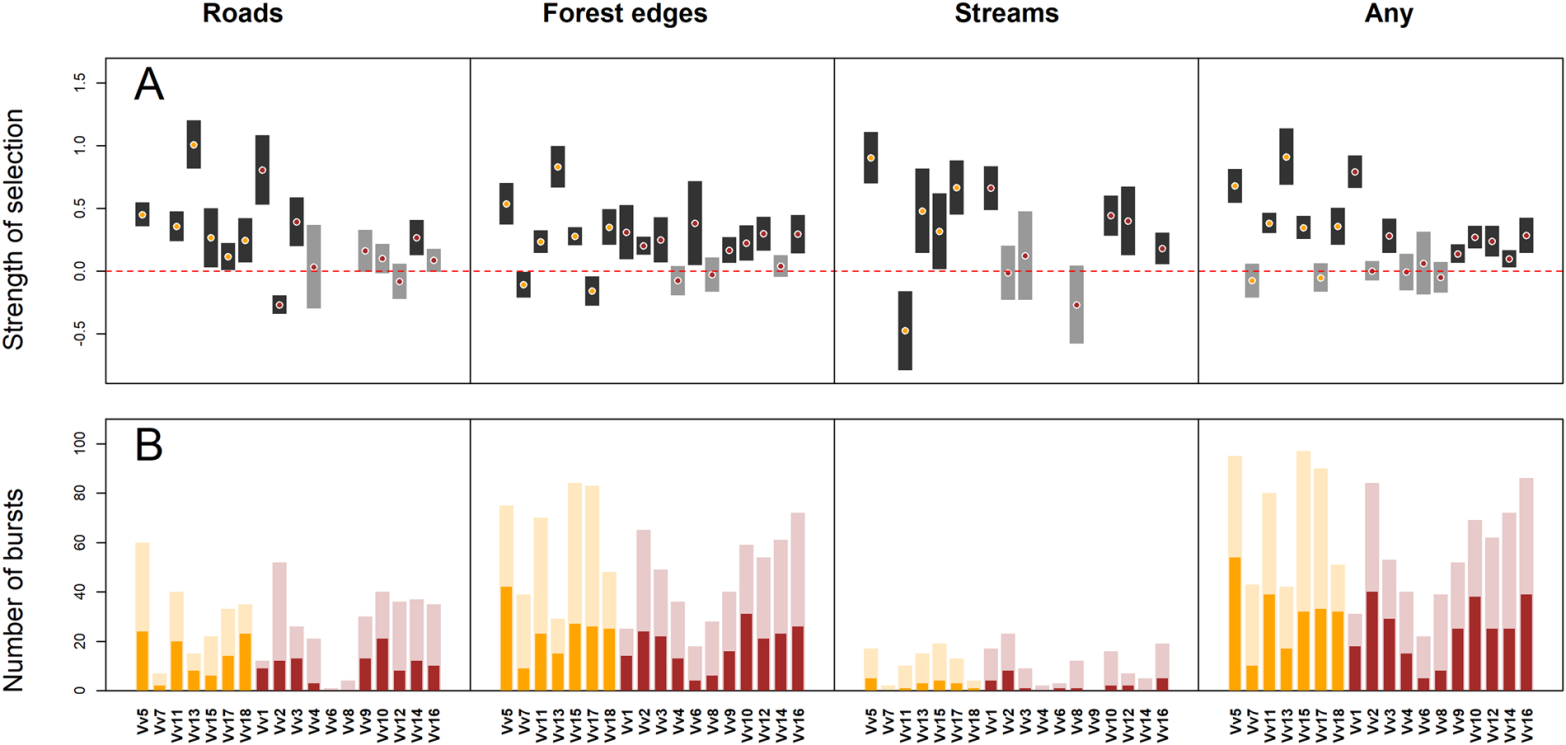
(**A**) Coefficients from the conditional logistic regression model assessing the propensity to remain in close proximity (<10 m) to a linear feature once it has been encountered (positive coefficient: selection; above the dashed horizontal line). Mean coefficient estimates are shown as dots (females: orange; males: brown) and vertical bars delineate the 95% confidence interval (black for coefficients significantly different from 0; grey for coefficients that did not differ from 0 at the α level of 0.05). (**B**) Number of bursts with linear feature tracking (LFT) events (dark bars; orange: females; brown: males) out of the total number of bursts with encounters of linear features (light bars). Burst counts from individual linear feature types do not add up to counts associated with the combination of all linear features (“any”) because linear features often co-occur. Individual IDs are shown on the x-axis of panel B and are linked with individual-based information provided in Supplementary Table S1.

**Figure 6:**
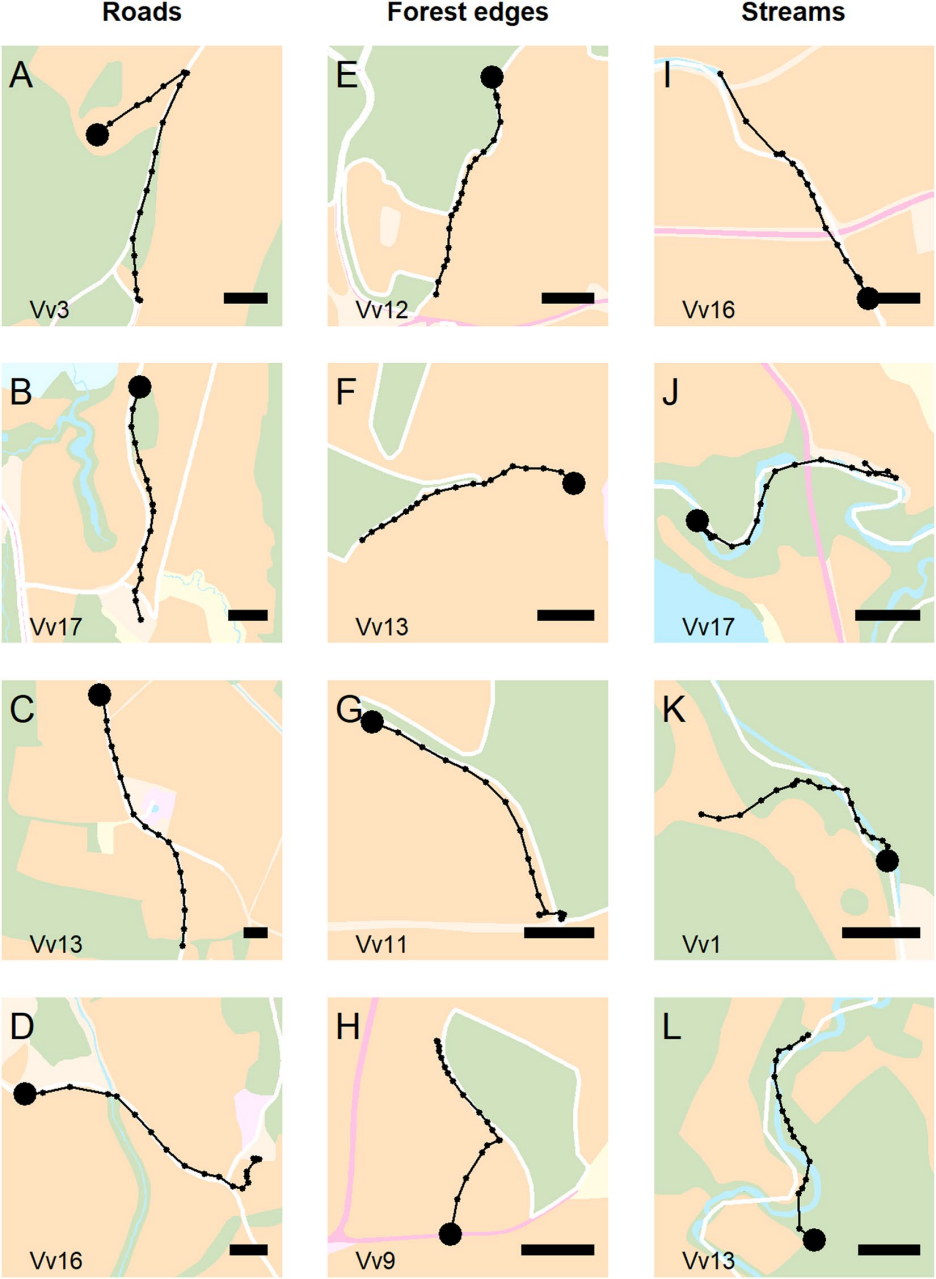
Examples of linear feature tracking (LFT) made apparent by high frequency GPS bursts. Background colors represents paler versions of the land cover classification described in Fig. 2. Focal linear features are indicated as white bands. Burst trajectories are shown as black lines. Black dots are GPS positions, the largest one indicating the start of the burst. Individual IDs (see also Supplementary Table S1) and a 50 m scale bar are provided in the lower left and right corners of each map, respectively. Maps were created in R version 3.5.1^44^ (www.R-project.org).

Conditional logistic regression in combination with a path-level null model, yielded significant positive coefficients associated with roads (9 of 15 individuals with 5 or more bursts with linear feature encounters, Fig. 5A), forest edges (13 of 18 individuals, Fig. 5A) and streams (8 of 12 individuals, Fig. 5A). Negative coefficients, suggesting that prolonged proximity to linear features was avoided once they were encountered, emerged in four cases (roads: 1 individual; forest edges: 2 individuals; streams: 1 individual; Fig. 5A). Of bursts that encountered roads, forest edges, streams, or any linear features, 41%, 39%, 22%, and 43% resulted in LFT, respectively (Figs 5B, 6, Supplementary Table S2). LFT occurred also in fox/feature type combinations that were associated with non-significant or negative selection coefficients (Fig. 5A).

### Duration of LFT (${{\boldsymbol{t}}}_{{\boldsymbol{LFT}}}$)

**Figure 7:**
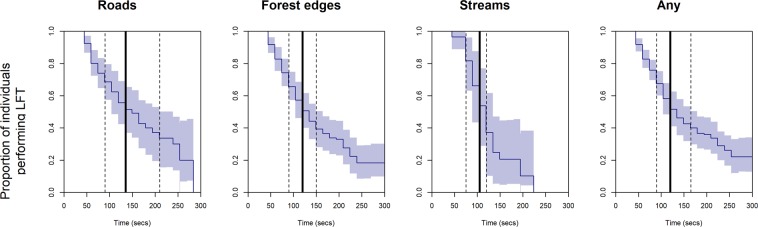
Empirical time-to-event curves (dark blue; 95% confidence band in lighter blue) for linear feature tracking (LFT) abandonment following commencement. Median times to abandonment (${t}_{{LFT}}$) are marked with solid vertical lines, together with their 95% confidence intervals (vertical dashed lines). Confidence bands were derived by bootstrapping, with sampling accounting for non-independence at both the individual and burst level. Curves do not drop below 1 until after 45 seconds have passed, due to the requirement of a minimum three positions within the proximity buffer (10 m) and the earliest event time (first position outside the proximity buffer) thus being 45 seconds.

Conditional on apparent LFT having commenced, median tracking times (i.e., the time by which 50% LFT events had not yet ended) were 135 s (95%CI: 90s–210s) for roads, 120 s (95%CI 90s–150s) along forest edges, and 105 s (95%CI: 75–120) for streams. Cox proportional hazards regression revealed that the risk of abandoning LFT along streams was significantly higher (i.e., more rapidly declining time to event curves) than along roads (coef = −0.43, exp(coef) = 0.65, SE = 0.26, z = −2.21 p = 0.03, Fig. 7), and tended to be higher compared with forest edges (coef = −0.32, exp(coef) = 0.73, SE = 0.19, z = −1.69, p = 0.09, Fig. 7).

### Movement speed during LFT (${{\boldsymbol{v}}}_{{\boldsymbol{LFT}}})$

**Figure 8:**
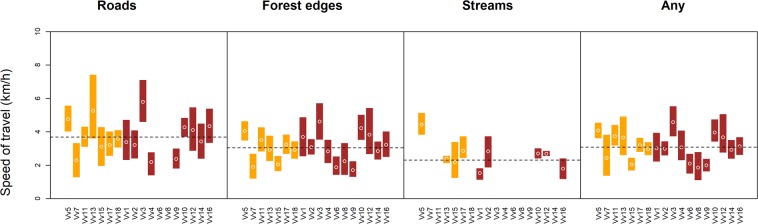
Average movement speed during LFT (${v}_{{LFT}}$). Points indicate mean coefficient estimates and vertical bars their associated bootstrapped 95% confidence intervals (females: orange; males: brown). Individual IDs for foxes are provided on the x-axis. Dashed horizontal lines indicate overall average LFT speeds for all individuals with at least two LFT events along a given linear feature type.

Average movement speeds were highest when foxes moved along roads (3.7 m/sec, range: 2.2–5.8 m/sec), followed by forest edges (3.1 m/sec, range: 1.7–4.62 m/sec and streams (2.31, range: 0.69–4.44), Fig. 8). Linear mixed regression indicated that log-transformed speeds during LFT along roads were significantly higher than LFT speeds along the other two feature types (forest edges: coef = −0.1, SE = 0.03, t = −3.25; rivers: coef = −0.32, SE = 0.08, t = −4.3). Speed during LFT also varied considerably among individuals. However, there seemed to be some consistency in the average speed at which an individual moved regardless of the type of linear feature (Fig. 8). For context, average observed distances associated with LFT events were 109 m (range across individuals: 72 m–211 m) along roads, 82 m along forest edges (49 m–1254 m), and 65 m (43 m–1114 m) along streams. These are observed distances limited by burst duration, without accounting for censoring, and should therefore be considered minimum distances.

## Discussion

Our study revealed pronounced linear feature tracking by red foxes in a highly fragmented landscape. High frequency GPS bursts and a path-level null model approach allowed us to detect fine-scale behavior that would have remained hidden had we employed conventional fix frequencies in the range of several minutes or hours. The incidence and strength of LFT varied among foxes and feature types. LFT was most pronounced in association with forest edges and roads (Fig. 5). The duration of LFT ($${t}_{{LFT}}$$) was shorter along streams than along roads and forest edges. Travel speeds ($${v}_{{LFT}})$$ were on average higher along roads than along forest edges and streams.

We found strong evidence of linear feature tracking in foxes, confirming our first prediction (Figs 5 and 6). The presence of linear features such as roads can have contrasting effects at different spatial scales. Individuals can avoid areas with high road density at large spatial scale (e.g., when establishing a home range^57^), but may select for them during daily activities at a smaller scale (e.g., moving^58^). Here we found that foxes selected roads at a very fine scale by performing LFT. Between 60% and 72% of foxes exhibited a propensity to remain in close proximity to linear features once they were encountered. Linear feature tracking events occurred even in fox/feature combinations that did not emerge with significant positive coefficients from the path analysis (Fig. 5). Overall, apparent linear feature tracking events occurred in 43% (95%CI: 37–48%) of bursts that came in proximity of either of the linear feature types used in our study.

While all foxes apparently tracked linear features occasionally, time-to-event analysis indicated that the median time until a linear feature was abandoned once LFT started was relatively short, 120 seconds (95%CI: 90s–165s) along the combination of all three linear feature types. Although longer LFT events also occurred, these were rare. The brief duration of LFT is likely attributable to a combination of species-specific and environmental characteristics. Our study area is highly fragmented, with an abundance of intersecting and tortuous linear features (Fig. 2). High levels of human disturbance/activity, including intensive hunting, the risk of intra-guild predation posed by Eurasian lynx *Lynx lynx*^59^ and a generalist foraging strategy of foxes, may further explain their variable and tortuous movement trajectories.

Using linear features, which are generally characterized by open habitat, allows foxes to move faster, as shown for wolves^10^. However, LFT may also make foxes more detectable to predators and hunters, and predation risk influences habitat selection^60,61^. The recurrent, but short-lived LFT exhibited by foxes may thus illustrate the fine scale behavioral decisions of a medium carnivore in a landscape of fear^62^, where foxes trade off ease of travel with predation and hunting risk.

Given the short duration of LFT by foxes in our study area, the phenomenon would likely remain hidden from telemetry studies employing conventional position fix rates in the order of hours or even several minutes. Our study was located in a fragmented, human-dominated landscape; other study systems with less tortuous linear features and more homogeneously structured landscapes^14^ may readily detect LFT using much longer inter and within burst intervals, perhaps even omitting bursts all together. We also expect that longer monitoring durations (months instead of days), accompanied by an increase in the total number of GPS positions, will improve detection of selection for linear features. Regardless of the study system, evidence of association within the individual home range (e.g., through conventional step selection analysis) is not a direct evidence of LFT, which requires that an animal’s path coincides with, not only periodically intersects, an underlying geographic feature. To detect true LFT, the temporal grain of the data must be fine enough to allow detection of intermittent deviations from a particular feature.

Developments in GPS tracking technology^63^ and the increasing availability of high-resolution spatial environmental data, have motivated a growing number of studies to employ position fix frequencies in the order of minutes, rather than hours or days, or a combination of both^64,65^. This provides opportunities to better understand the interactions between linear features and wildlife. For example, our approach allowed us to detect and quantify LFT and not only the selection for linear features^30,58^. In addition, our framework could be extended to help distinguishing the intrinsic and extrinsic drivers that prompt individuals to perform LFT. This would involve the use of individual and/or site specific covariates in the conditional logistic regression when quantifying LFT, or in the time to event analysis when quantifying time to departure from the linear feature.

LFT is not the only fine-scale behavior that may be missed or its prevalence underestimated even at moderately high telemetry fix frequencies. Clusters of positions are often used to locate and subsequently investigate specific behaviors such as resting^32^, foraging^66^, and to study kill rates^67^, inter-specific interactions^68^, or responses to human disturbance^32^. Some key behaviors may only take minutes or even seconds to execute, making them virtually undetectable with conventional telemetry schedules. For example, studies quantifying predation and its spatial determinants may be biased towards larger prey items that require longer handling times and thus are more likely to result in observable position clusters, whereas small prey may go undetected^69^. Similarly, high frequency position data and a null model approach could help quantify how infrastructure mitigation structures (such as wildlife crossings on roads) are used by wildlife and, assuming appropriate scope of the study, how this use may scale up to the level of landscapes and populations.

Being able to obtain fine scale data, “may give us the potentially false impression that fine spatial and temporal scale dynamics are relevant to ecology or, most critically, conservation”^70^. We argue that LFT, or other behavior that may be so short-lived that GPS intervals lasting seconds or minutes are needed to detect and properly describe it, are relevant because they can have key impacts on life history and populations in general. For example, such behaviors can be associated with significant spikes in foraging opportunities, which in turn can be important to maintain population density^71^. Even if linear features are not selected for or are actually avoided within the home range, animals may utilize them once they are encountered. Overall, in highly fragmented human-impacted landscapes such as our study area, even random walk-type movement or moderate avoidance would lead to frequent encounters of linear features and thus opportunities for interactions with them at fine scales. In such fragmented scenarios, fine temporal and spatial scales can help us better understand the mechanisms involved in wildlife behavioral responses, which in turn can help predict the effects of human-induced environmental change on species and communities^72^. The increasing availability of fine scale environmental information, such as the detailed land cover maps used in this investigation, also boosts the utility of and opportunity to use fine scale movement data^73,74^.

Managing the tradeoff between resolution and longevity is an ubiquitous challenge in wildlife telemetry^33,75^. Configuring GPS fix schedules into a sequence of bursts provides a practical way to balance the need for fine-scale information about animal movements with the simultaneous desire for home-range level inferences. Technical characteristics of wildlife GPS telemetry devices and the goals of a given study will guide the choice of inter-burst interval and the number and frequency of position fixes within bursts. For increased utility and power efficiency, the initiation of bursts could be made conditional on times, positions, or inferred activity, with the help of schedules, proximity rules (e.g., geo-fencing), and integrated accelerometer data. Even in cases where scheduling is not constrained by other considerations, GPS position accuracy will provide a lower limit for the scale at which inferences can be drawn^34^. We concede that, although bursts provide a reasonable tradeoff between the desire to obtain position information at both fine and coarser scales, investigators should be cautious when designing telemetry schedules. Study designs well-suited for targeting one or a few questions are generally preferable over designs that attempt to answer many questions at once. The main goal of the study should determine the most appropriate position fix interval^34,35^, within the overall constraint of battery life.

Finally, technical and analytical developments in peripheral devices integrated into wildlife GPS collars may allow for inferences about behavior at fine scales, without the need for very high GPS fix rates. Accelerometers can be used to prompt periods of higher fix rates^33^ or to reconstruct detailed movement paths^76^. Similarly, telemetry collars with integrated photo and video cameras could help detect direct evidence of fine-scale behavior such as LFT. For instance, intensive fieldwork is necessary to study prey selection and kill rates of carnivores^77^, and neck-mounted cameras help estimate kill rates accurately^78^.

## Conclusions

Linear feature tracking, especially in carnivores, is frequently mentioned, but has rarely been tested for or quantified as such. In our study, the use of GPS bursts, in combination with a path-level null model approach, allowed us to detect and quantify linear feature tracking occurring at a fine spatial and temporal scale in a highly fragmented landscape. We also detected individual variation in the propensity to perform LFT; we encourage further studies to determine potential individual and environmental drivers of this variation. While the importance of fine-scale data may at times be overemphasized^70^, increasing fragmentation in human-dominated environments may require finer-scale movement decisions, and, in turn, observations of sufficiently high spatio-temporal resolution. Another requirement is the availability of analytical procedures that do not only account for the autocorrelation inherent in fine scale data, but make use of the signal about underlying structure contained therein^52^. Further developments in telemetry technology and its application, together with flexible analytical approaches, will help study behaviors that are manifested over very short durations, yet are biologically significant.

## Supplementary information

Supplementary information

## Data Availability

Data used in this analysis are available from the corresponding author upon request.
