# Supplementary material for: High frequency GPS bursts and path-level analysis reveal linear feature tracking by red foxes
Source: Sci Rep. 2019 Jun 20;9:8849. doi: 10.1038/s41598-019-45150-x (PMC6586955; doi:10.1038/s41598-019-45150-x)
Supplement: Supplementary file 1 — Supplementary information [file 41598_2019_45150_MOESM1_ESM.pdf]

Supplementary Information for "High frequency GPS bursts and path-level analysis reveal linear feature tracking by red foxes"

Richard Bischof[1], Jon Glenn Omholt Gjevestad[2], Andrés Ordiz[1], Katrine Eldegard[1], Cyril Milleret[1]

[1]Faculty of Environmental Sciences and Natural Resource Management, Norwegian University of Life Sciences, PO Box 5003, NO-1432 Ås, Norway

[2]Faculty of Science and Technology, Norwegian University of Life Sciences, PO Box 5003, NO-1432 Ås, Norway

Supplementary Table S1: Individual attributes of 18 red foxes captured and fitted with GPS collars to capture fine-scale movement behavior. Information related to GPS tracking is presented as the number of consecutive days that yielded GPS data (data days) used in the analysis, and the number of fixes recorded. The number of moving positions represents the number of GPS positions that were part of bursts collected while the animal was travelling (see Methods section "*GPS tracking and data preparation"*). Age category "juvenile" refers to individuals caught and GPS-collared during their year of birth, based on their physical appearance. Due to difficulties determining ages of older individuals without more invasive methods, all other individuals were categorized as "adult", but may have included individuals <1 year of age (e.g. during late winter, following the year of birth).

ID	Sex	Age category	Body weight (kg)	Date collared	Data days	Number of positions	Number of moving positions
Vv1	male	adult	7.9	1/18/2018	6.2	1994	965
Vv2	male	adult	6.2	2/10/2018	10.9	3267	1864
Vv3	male	adult	6.4	2/21/2018	4.6	2939	1734
Vv4	male	adult	5.9	2/22/2018	8.9	3737	1507
Vv5	female	adult	5	4/14/2018	10.8	3899	2724
Vv6	male	juvenile	4.6	7/27/2018	13.1	2154	1215
Vv7	female	juvenile	3.5	7/27/2018	11	2906	1646
Vv8	male	juvenile	4.9	8/3/2018	11.5	3331	1671
Vv9	male	juvenile	4.6	9/5/2018	12.9	4319	1523
Vv10	male	juvenile	4	9/7/2018	11	3712	1815
Vv11	female	juvenile	4.1	9/21/2018	9.2	4617	2344
Vv12	male	adult	6.5	11/12/2018	14.2	3298	2038
Vv13	female	adult	6.4	12/8/2018	7.9	3041	1428
Vv14	male	adult	5.5	12/9/2018	16.4	2519	2002
Vv15	female	adult	5.5	12/13/2018	27.8	3643	2448
Vv16	male	adult	5.5	1/17/2019	20.5	4195	2014
Vv17	female	adult	4.6	3/10/2019	27	4371	2576
Vv18	female	adult	4.3	3/24/2019	14.3	1123	1054

Supplementary Table S2: Summary of average counts of linear feature (LF) encounters and linear feature tracking (LFT) events. Tallies are shown at the event level (number of linear feature encounters and LFT events) and at the burst level (number of bursts with linear feature encounters and LFT events). The distinction between event and burst level tallies is made because multiple LFT encounters (and thus LFT events) are possible within the same 20-position GPS burst, i.e. when the trajectory formed by the sequence of positions within the bursts first exits and then re-enters the distance threshold buffer associated with the linear feature. Bootstrapped 95% confidence intervals are provided in parentheses. The average number of GPS bursts per individual was 100.6 (95% CI: 88.3-110.6)

	**Event level**			**Burst level**		
**Feature type**	**Average number of LF encounters**	**Average number of LF events**	**Proportion**	**Average number of bursts with LF encounters**	**Average number of bursts with LFT events**	**Proportion**
Roads	39.6 (28.0- 47.8)	13.2 (9.9-16.8)	0.32 (0.26-0.41)	28.1 (21.2-34.9)	12.4 (9.6-15.3)	0.41 (0.34-0.47)
Forest edges	83.3 (69.6- 98.7)	22.0 (17.3-25.6)	0.26 (0.23-0.29)	51.9 (42.1-60.5)	20.4 (15.6-23.9)	0.39 (0.34-0.43)
Streams	16.0 (11.6- 21.0)	3.1 (2.1- 4.1)	0.17 (0.13-0.20)	11.4 (8.6-14.4)	2.9 (1.9- 4.3)	0.22 (0.18-0.25)
Any feature	103.1 (84.4-117.7)	29.5 (23.7-35.5)	0.28 (0.25-0.31)	61.6 (49.6-72.1)	26.9 (21.5-31.4)	0.43 (0.37-0.48)
